# Unexpected early loosening of rectangular straight femoral Zweymüller stems with an alumina-reduced surface after total hip arthroplasty—a prospective, double-blind, randomized controlled trial

**DOI:** 10.1186/s10195-023-00743-1

**Published:** 2024-03-02

**Authors:** Céline S. Moret, Salim El Masri, Benjamin L. Schelker, Niklaus F. Friederich, Michael T. Hirschmann

**Affiliations:** 1 Department of Orthopaedic Surgery and Traumatology, Kantonsspital Baselland, CH-4101 Bruderholz, Switzerland; 2 Department of Orthopaedic Surgery and Traumatology, Rhön Klinikum Campus, 97616 Bad Neustadt an Der Saale, Germany; 3 Department of Orthopaedic Surgery and Traumatology, University Hospital Basel, Basel, Switzerland

**Keywords:** Total hip arthroplasty, Uncemented stem, Aluminium oxide grit blasting, Acid etching, Dual-energy x-ray absorptiometry, Survivorship

## Abstract

**Background:**

Alumina particles from the grit blasting of Ti-alloy stems are suspected to contribute to aseptic loosening. An alumina-reduced stem surface was hypothesized to improve osseointegration and show comparable short-term outcomes to those of a standard stem.

**Methods:**

In this prospective, double-blind, randomized trial, 26 standard (STD) and 27 experimental new technology (NT) stems were implanted. The latter were additionally treated by acid etching and ice blasting to remove alumina particles from the grit-blasting process. Follow-up occurred at 12 and 24 months. Bone mineral density (BMD) around the stem was measured by a dual-energy x-ray absorptiometry device (DEXA). Radiographs were reviewed for alterations. Clinical scoring comprised the Western Ontario and McMaster Universities Osteoarthritis Index (WOMAC) and the Harris Hip Score (HHS). Survival rates were calculated up to 50 months.

**Results:**

Lower mean BMD and more severe cortical hypertrophies were found in the NT group. At 12 months, radiolucent lines were observed mostly in the metaphyseal zone for both groups, with a progression tendency in the NT group at 24 months. At 12 months, pain scores and the WOMAC total and physical activity scores were significantly lower in the NT group, without any differences thereafter. The number of NT stem revisions amounted to 6 (24%) and 11 (41%) at 24 and 50 months, respectively.

**Conclusion:**

In the NT group, unexpected catastrophic failure rates of 41% caused by early aseptic loosening were noted within 50 months. Compared with the STD stems, NT stems lead to poor clinical and radiographic results.

*Level of evidence*: II.

*Trial Registration*: NCT05053048.

## Introduction

Total hip arthroplasty (THA) using an uncemented straight stem with rectangular cross section is a well-established option for hip surgeons [1–3]. Approximately 40 years ago, the Zweymüller Alloclassic stem—a conical-rectangular straight, tapered stems with a rectangular cross section—was introduced into the global market [4]. It provides firm primary and rotational stability through direct press-fit fixation of the four corners in the cortex and cancellous bone of the femoral metaphysis and diaphysis, as well as a better preservation of the endosteal blood supply by not filling the entire medullary cavity [4].

It is commonly accepted that the implant surface plays a decisive role for osseointegration. One method to create an adequate surface roughness of 4–6 μm is the corundum, also called aluminium oxide (Al_2_O_3_) or alumina, grit-blasting process of the stem, which allows bone in- and ongrowth, and provides reliable biologic long-term fixation [5, 6]. However, residual alumina particles used for the grit-blasting process are thought to invade the periprosthetic tissues and cause metal wear of the endoprosthetic surface as well as third body wear [7–10]. Alumina and metal wear particles are known to contribute to aseptic loosening, a major cause for hip revision arthroplasty [8–13]. Aluminium ions are also suspected to impair bone formation by a possible competitive action to calcium ions [14].

To remedy this problem, a Zweymüller stem, called new technology (NT), stepless (SL)-PLUS, with an alumina reduced surface was developed a decade and a half ago. This new stem had the same geometry as the standard (STD) SL-PLUS Zweymüller stem but differed only in terms of surface treatment after grit blasting. Hence, a short-acid etching with hydrofluoric acid (HF) followed by dry ice blasting were undertaken to remove alumina particles of the grit-blasted titanium alloy implants. This method can achieve an alumina particle reduction of up to 96% without changing the surface roughness [15].

The aim of this study was to compare the NT-SL-PLUS Zweymüller stems with the STD-SL-PLUS Zweymüller stems, in terms of (1) bone mineral density (BMD) around the stem as a surrogate for osseointegration, (2) radiological alterations and clinical outcome, and (3) survival rates of the implant. It was hypothesized that NT stems would show at least comparable short-term (24 months) radiological and clinical outcomes as the STD stems.

## Material and methods

### Study design and population

This study was approved by the local ethical committee (EKNZ no. 254/05). Written informed consent was obtained from all patients willing to participate. The procedures used in this study adhere to the tenets of the Declaration of Helsinki.

**Fig. 1:**
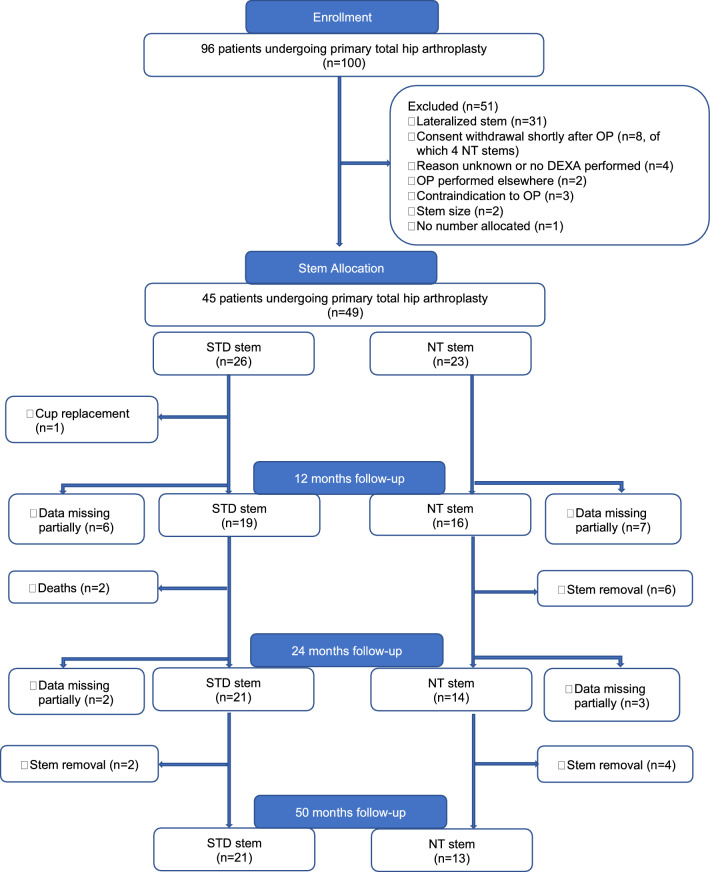
Patient flow chart

From August 2006 to September 2008, patients aged 18 years and older undergoing primary THA for primary or secondary osteoarthritis were enrolled in this prospective, randomized, double-blind, controlled clinical trial conducted in a university affiliated hospital. Exclusion criteria were ongoing therapy with cortisone or medications influencing bone metabolism. Further exclusion criteria after enrolment can be seen in the patient flow chart (Fig. 1).

Eligible patients were randomly assigned (1:1) to receive either an uncemented NT-SL-PLUS Stem (Smith&Nephew Orthopaedics AG, Aarau, Switzerland) or a matching uncemented STD-SL-PLUS stem (Smith&Nephew Orthopaedics AG, Aarau, Switzerland) in a blinded fashion. Both patients and surgeons performing the THAs and assessing follow-up outcomes were blinded to the stem assignment. For stem allocation, block randomization with block size 10 was applied.

Both rectangular SL straight stems were made of titanium alloy (Ti-6Al-7Nb). The control stem (STD SL-PLUS) and the experimental stem (NT SL-PLUS) underwent alumina grit blasting to reach the adequate roughness of 4–6 μm. The surface of the NT SL-PLUS stem was thereafter additionally treated chemically by short-acid etching with HF and mechanically by dry ice blasting to loosen and remove the residual alumina particles up to 96% without changing the existing surface microtopography.

Of the 96 patients initially enrolled, four patients who had received an NT stem withdrew consent shortly after surgery (Fig. 1). Eventually, 45 patients undergoing a total of 49 primary THAs were included in this study. The mean age at surgery was higher in the STD group (71 years) than in the NT group (64 years). Mean body mass index (BMI), sex, and operation side were equally distributed. Patient demographics are represented in Table 1.

**Table 1:** Patient baseline demographics

Variables at surgery	STD stem *n* = 26	NT stem *n* = 23
Mean age, years (SD)	70.9 (8.9)	64.2 (9.2)
Mean BMI, kg/m^2^ (SD)	28.3 (6.7)	27.0 (6.3)
Sex, *n* (%)		
Female	17 (65)	14 (61)
Male	9 (35)	9 (39)
Side, *n* (%)		
Left	13 (50)	11 (48)
Right	13 (50)	12 (52)

*BMI* body mass index, *kg* kilogram, *m* meter, *n* number, *NT* new technology, *SD*: standard deviation, *STD* standard

### Procedure and clinical Outcomes

The THAs were performed by one senior, board-certified orthopedic surgeon with more than 25 years of experience. Preoperative planning with the mediCAD software (Altdorf/Landshut, Germany) and intraoperative adjustments were carried out. An anterolateral approach was used.

**Fig. 2:**
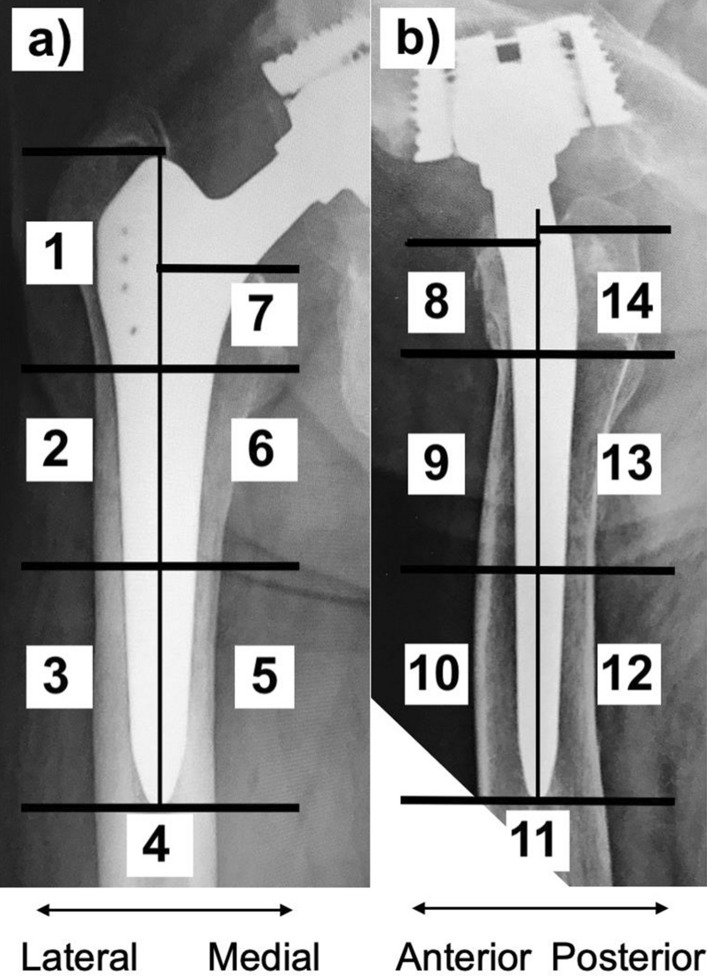
Representation scheme of Gruen zones 1–7 on an anteroposterior view (**a**) and 8–14 on an axial view (**b**) of a hip radiograph

BMD around the stem was measured as a surrogate for prosthesis in-growth by a dual-energy x-ray absorptiometry device (DEXA) (General Electric) on an anteroposterior view, according to the Gruen zones (zones 1–7) as localization scheme (Fig. 2) [16]. Measurements were performed 7 days (baseline) after surgery and at 3, 6, 12, and 24 months postoperatively. For each Gruen zone, the mean BMD value and the mean difference in BMD between the baseline and the different follow-up times were assessed.

Standard conventional anterior–posterior and lateral hip radiographs were reviewed for alterations at 12 and 24 months postoperatively. Thus, radiolucent lines (RLs), osteolysis, hypertrophies, and atrophies around the stem were assessed according to Gruen et al. [16] (zones 1–14) (Fig. 2). These were classified as minimal, moderate or severe when 1 mm, 2 mm, or > 2 mm in width, respectively. Additionally, heterotopic ossifications were noted, according to Brooker et al. [17]. Stem subsidence was also measured.

For clinical scoring, the Western Ontario and McMaster Universities Osteoarthritis Index (WOMAC) and the Harris Hip Score (HHS) were assessed. The WOMAC score is a self-administered 24-item questionnaire that assesses pain, stiffness, and physical function of the affected hip joint. Higher scores indicate a better performance. The 11 items of the HHS are grouped into three sections: pain, function/everyday activities, and range of motion. The range of motion was measured for abduction, adduction, external rotation, and flexion. Baseline HHS and WOMAC were evaluated preoperatively. Both scores as well as patient satisfaction, evaluated on a four-level Likert scale ranging from not satisfied to very satisfied, were assessed at 12 and 24 months postoperatively.

Furthermore, THA survival rates and adverse events such as aseptic or septic loosening, infections, periprosthetic fractures, and implant failure were documented up to 50 months after THA, despite an initial study period of 24 months.

### Statistical analysis

A sample size of 20 patients in each group was calculated to detect expected differences in BMD as well as radiological and clinical outcomes. With an estimated 20% of dropouts, a sample size of 25 patients per group was opted for.

Data were analyzed by a professional statistician using SAS (Version 9.1, SAS Institute Inc., Cary, NC, USA) and Statistica (Version 9, TIBCO Software Inc., Palo Alto, CA, USA). Descriptive statistics are presented with mean and standard deviation (SD) and frequency counts and percentages for categorical variables. Normality of continuous variables was evaluated with the Kolmogorov–Smirnov test and between-group comparison for radiographic alterations and clinical outcomes (WOMAC, HHS) with an independent sample *t*-test. Two further different mixed models, one with absolute BMD values and a second one with baseline BMD changes after surgery were used for the BMD measurements between groups.

Finally, the cumulative incidence of survivorship after primary THA was calculated at 12, 24, and 50 months with stem revision as an endpoint. Normal data is reported as mean (SD) and nonnormal as median (range). Results are presented with a 95% confidence interval (CI). For all analyses, *p* < 0.05 was considered as statistically significant.

## Results

### DEXA findings

**Fig. 3:**
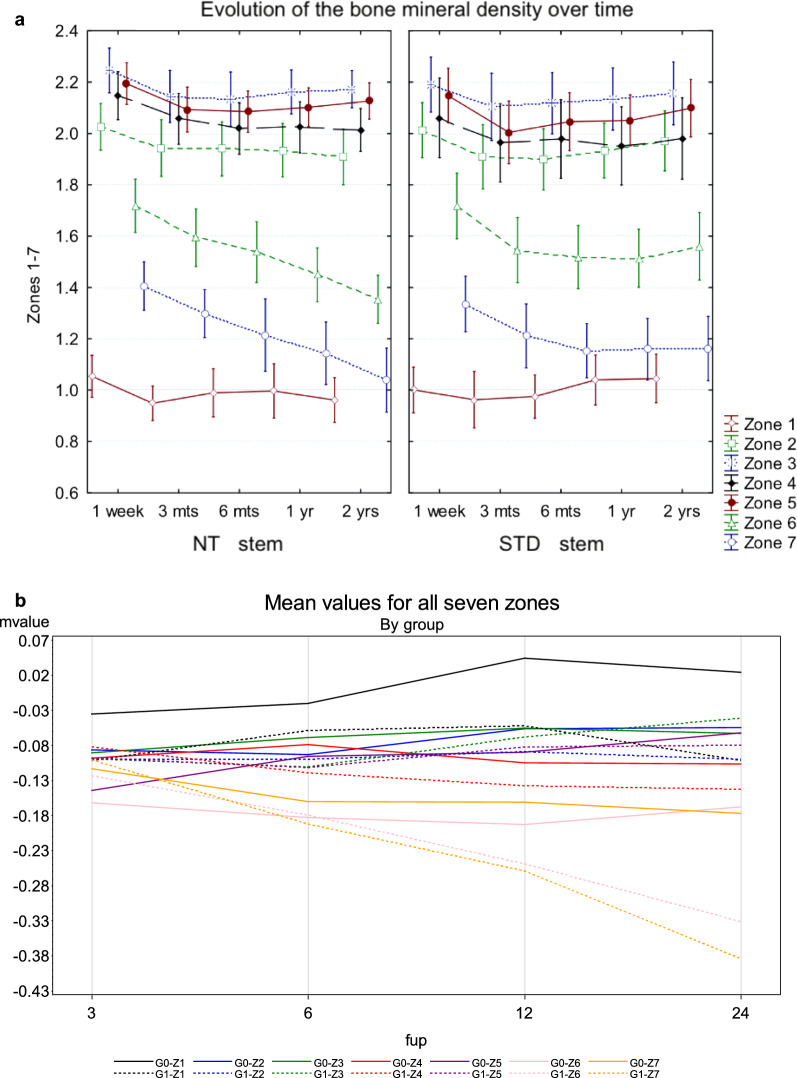
**a** Evolution of the mean bone mineral density (BMD, in g/cm^2^, including 95% confidence interval) in the different Gruen zones over time for the NT and the STD stem. **b** Plot of raw mean bone mineral density (BMD) differences over time from 3 to 24 months postoperatively, grouped in Gruen zone and stem cohort. Abbreviations: *fup* follow-up in months, *G0* STD stem, G1 NT stem, *Z1-7* Gruen zones 1–7, *mvalue* mean value

After a small decrease of mean BMD per zone, stabilization of mean values was noted from 3 months on for almost all zones in both groups, except for the medial metaphysis and the most proximal diaphysis region (zones 6 and 7) of the NT group, where a significantly lower BMD (*p* = 0.006) was measured at 12 and 24 months follow-up compared with the STD group (Fig. 3a). Concerning the significant mean change of BMD comparing the NT and STD groups in zones 6 and 7, there is a higher negative difference for the NT group (*p* = 0.0002) beginning 12 and 6 months postoperatively, respectively (Fig. 3b). Leading to an additional drop in BMD of 16% and 20% for zones 6 and 7, respectively at 24 months.

### Radiographic findings

At 12 months follow-up, no heterotopic ossification grades II, III, or IV were observed regardless of the implanted stem. However, 15% of the NT group showed grade I ossification compared with 30% in the STD group. An increase in heterotopic ossification was seen in both groups at 24 months follow-up: 40% grade I, 10% grade II, and 5% grade III in the NT group compared with 57% grade I and 5% grade II in the STD group.

In the NT group, five cases of cortical hypertrophy were detected at 12 months and an additional four at 24 months postoperatively, compared with four and six, respectively, in the STD group. At 24 months follow-up, more than 10% of the stems showed in the NT group signs of moderate and severe hypertrophy in zones 2, 3, 5, and 6, whereas the STD group showed only minimal to moderate hypertrophy.

**Fig. 4:**
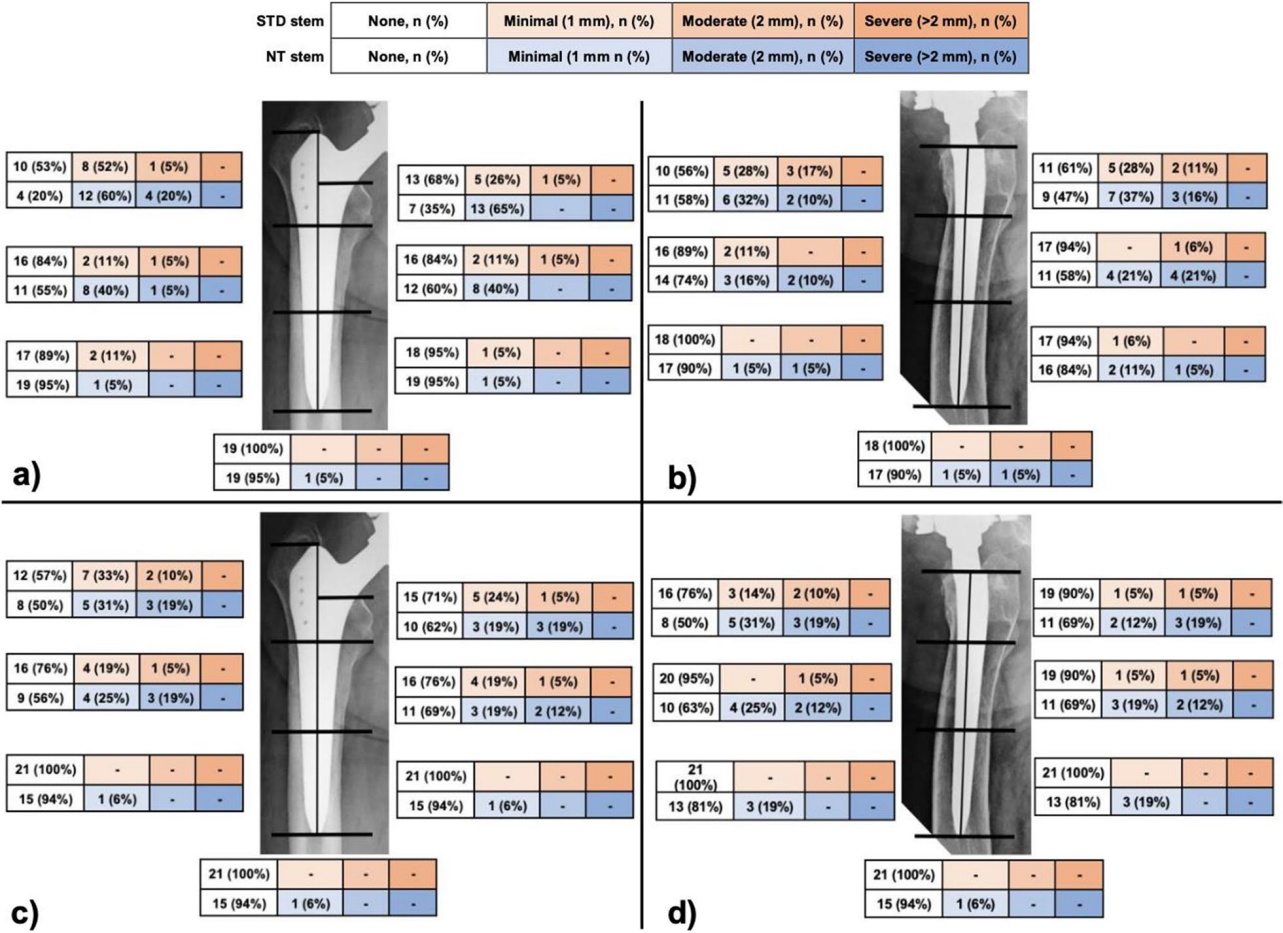
Comparison of radiolucent lines (RLs) by Gruen zones between standard (STD) stem group and the new technology (NT) stem group. Zones 1–7 on an anteroposterior view at 12 months (**a**) and at 24 months (**c**) follow-up. Zones 8–14 on an axial view at 12 months (**b**) and 24 months (**d**)

At 12 months follow-up, RLs were found mostly in zones 1 and 7 in up to 80% and 65% of the NT group, respectively, compared with 47% and 32% of the STD group, respectively. At 24 months follow-up, all NT stems had developed additional RLs, (zone 1, 2, 6, 7, 8, 9,13, and 14) with percentages ranging from 31% to 50%, in contrary to the STD group (Fig. 4).

After 12 months, two (10%) cases of osteolysis were described in the NT group and another case arose 24 months postoperatively. In the STD group, three (16%) showed osteolysis at 12 and none at 24 months.

A total of two stem subsidences were reported in the NT group. One NT stem subsided 4 mm at 12 months and another one 5 mm at 24 months postoperatively, compared with none in the STD group.

### Clinical outcomes

At 12 months follow-up, WOMAC total score, as well as the subsection “pain” and “physical activity” were significantly lower in the NT group. In the STD group, only the HHS subscore of pain was significantly higher. There were no significant differences at 24 months follow-up in either WOMAC score or HHS after exclusion of six revision cases in the NT group (Table 2). Patient satisfaction is presented in Table 3.

**Table 2:** WOMAC and HSS at 12 and 24 months

	Preoperative		12 months follow-up		24 months follow-up	
	STD stem *n* = 26	NT stem *n* = 23	STD stem *n* = 21	NT stem *n* = 20	STD stem *n* = 20	NT stem *n* = 15
	Mean (SD)	Mean (SD)	Mean (95% CI)	Mean (95% CI)	Mean (95% CI)	Mean (95% CI)
Harris hip score						
Total score	54.8 (± 11.9)	57.2 (± 14.3)	94.0 (90.1–97.9)	83.3 (73.4–93.2)	95.4 (92.4–98.4)	95.3 (89.8–100.8)
			*p* = 0.13		*p* = 0.70	
• Pain score	15.5 (± 6.7)	16.3 (± 8.3)	41.9 (39.7–44.0)	33.4 (26.2–40.5)	43.0 (41.5–44.4)	40.4 (36.4–44.5)
			*p* = 0.70		*p* = 0.70	
• Function gait score	30.8 (± 6.26)	32.5 m(± 7.4)	43.2 (40.7–45.6)	41.0 (37.4–44.6)	43.5 (40.6–46.4)	45.9 (44.4–47.5)
			*p* = 0.44		*p* = 0.33	
• Absence of deformities	3.95 (± 0.21)	3.84 (± 0.37)	4.00	4.00	4.00	4.00
• Range of motion	4.66 (± 0. 32)	4.62 (± 0. 47)	4.9 (4.9–5.0)	4.9 (4.8–5.1)	4.9 (4.9–5.0)	4.9 (4.9–5.0)
			*p* = 0.78		*p* = 0.72	

	Preoperative		12 months follow-up		24 months follow-up	
	STD stem *n* = 26	NT stem *n* = 23	STD stem *n* = 21	NT stem *n* = 20	STD stem *n* = 20	NT stem *n* = 15
	Mean (95% CI)	Mean (95% CI)	Mean (95% CI)	Mean (95% CI)	Mean (95% CI)	Mean (95% CI)
WOMAC						
Total score	48.8 (43–54)	49.0 (41–56)	96.3 (93–99)	84.5 (76–93)	95.6 (91–100)	92.8 (86–100)
			*p* = 0.02		*p* = 0.54	
• Pain	53.8 (48–60)	46.8 (39–54)	97.6 (95–100)	84.3 (75–93)	98.8 (96–101)	95.0 (90–100)
			*p* = < 0.01		*p* = 0.08	
• Stiffness	50.5 (41–60)	50.6 (41–60)	96.4 (93–100)	84.4 (73–96)	96.3 (92–100)	94.2 (87–101)
			*p* = 0.09		*p* = 0.87	
• Physical activity	47.1 (41–53)	45.6 (41–57)	95.8 (92–99)	84.6 (76–94)	94.6 89–100)	92.0 (84–99)
			*p* = 0.049		*p* = 0.57	

*CI* confidence interval, *n* number, *na* not available, *NT* new technology, *SD* standard deviation, *STD* standard, *WOMAC* Western Ontario and McMaster Universities Osteoarthritis Index

**Table 3:** Patient satisfaction at 12 and 24 months

	12 months follow-up		24 months follow-up	
	STD stem *n* = 21	NT stem *n* = 17	STD stem *n* = 21	NT stem *n* = 15
Very satisfied *n* (%)	18 (86)	13 (65)	19 (91)	13 (81)
Generally satisfied *n* (%)	3 (14)	3 (15)	2 (9)	2 (13)
Partially satisfied *n* (%)	None	3 (15)	None	1 (6)
Not satisfied *n* (%)	None	1 (5)	None	None

*n* number, *NT* new technology, *STD* standard

### Survival rate

**Fig. 5:**
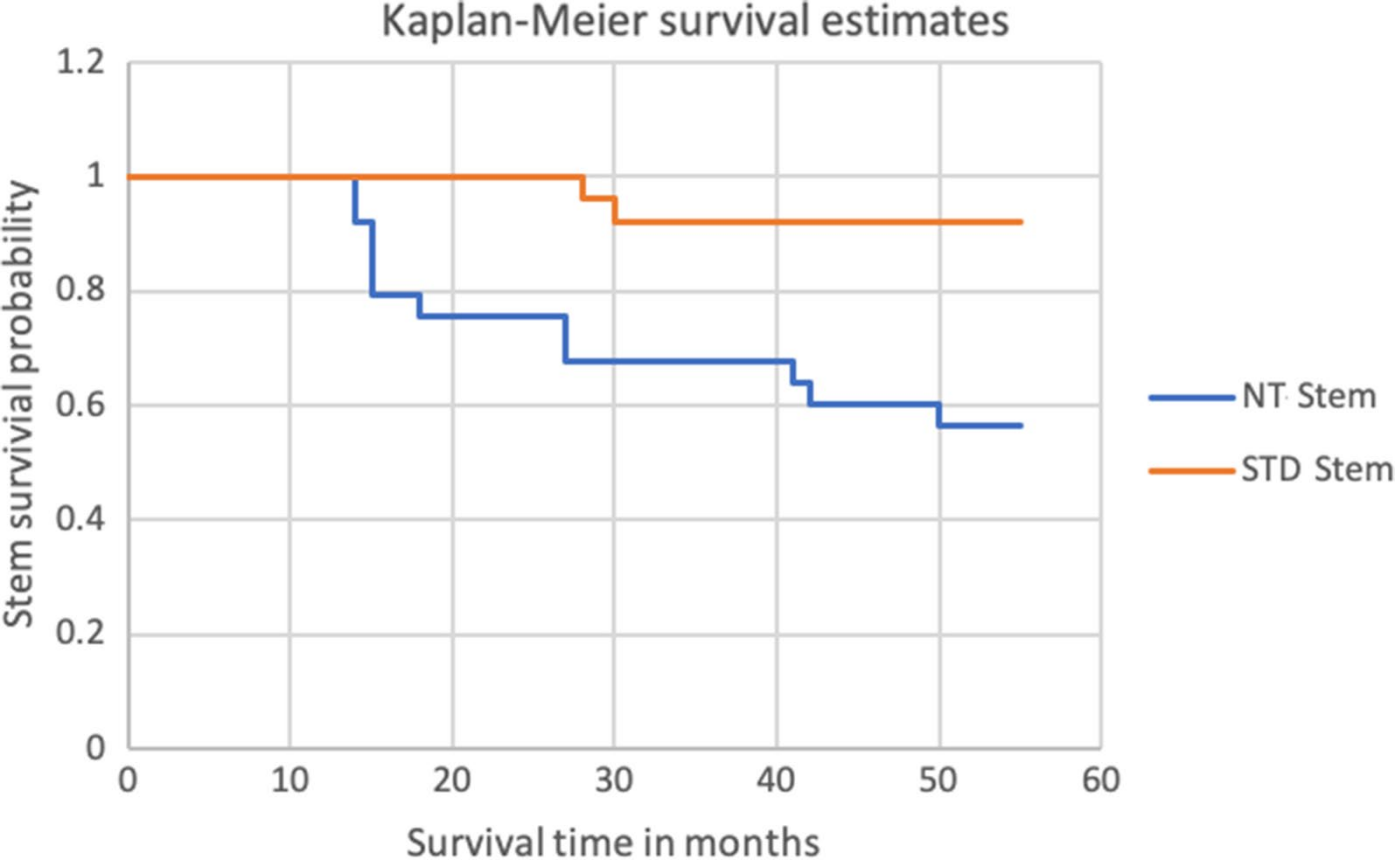
Kaplan–Meier survival estimates for the implanted stems up to 55 months after primary THA. Two standard (STD) stems failed at a mean of 29 months and 11 (41%) of the 27 implanted new technology (NT) stems had failed within 50 months

Within the initial study period of 24 months, six of the included NT stems were revised but none before 12 months. Thus, the cumulative incidence for stem failure at 24 months in this group was 24%. Finally, after extension of the observation period to 50 months due to the aforementioned results, a further four NT stems were revised. Including four patients who were not included in the original study, of which one NT stem failed, the total number of revisions amounts to 11, respectively 41% (11/27) of all implanted NT stems. The reasons were aseptic loosening in ten and infection in one case. The median time to revision was 18 months (range 14–50 months). On the other hand, two (7.7%) STD stems failed due to aseptic loosening and were revised at a mean of 29 months. The Kaplan–Meier survival estimates for both stem groups are shown in Fig. 5.

## Discussion

The major finding of this study was that the implantation of a rectangular femoral stem with an Al_2_O_3_-reduced surface in THA led to unexpected catastrophic failure rates, almost exclusively due to early aseptic loosening. Given these results, it is our ethical duty to report these adverse events to avoid any further potential harm to patients until more conclusive findings concerning this surface treatment are obtained, at least for use in THA.

Analyzing the results of the study, different potential reasons for early loosening must be considered. Long-term stability of the Zweymüller stem relies on its distal anchoring in the diaphysis of the femur [18]. Thus, a distal cortical hypertrophy is frequently seen in uncemented stems and is not thought to affect the clinical outcome or stem stability [19–21]. In the present study, more cases of cortical hypertrophy were noted at 2 years in the NT group than in the STD group, especially in the diaphysis around the stem (zones 2, 3, 5, 6). This can be interpreted as a response of the cortical bone to the unsatisfactory osseointegration eventually leading to instability and abnormal load transmission from the stem to the bone [19–21]. Patients with NT stems developed slightly less but more severe heterotopic ossification at 2 years follow-up; however, this finding is not believed to be of clinical relevance.

Almost half of patients with an STD stem had RLs present in metaphyseal zones 1 and 7 at 12 months postoperatively, but without progression at 24 months. These data build on the study by Zweymüller et al. [22], who analyzed the extent of RLs in 95 patients with a Zweymüller Alloclassic cementless stem 6 years after surgery. The authors found RLs in 45% of the patients almost all RLs in Gruen zones 1 and 7 with no progression in number and thickness at 10 years. Based on both the observation of Zweymüller et al. [22] and the present study, this suggest that RLs are a frequent finding and are thought to appear early (< 1 year) after THA with a STD stem. Furthermore, if their presence remains unchanged, the stability of the implant should not be jeopardized [22]. In contrast, the NT group of the present study showed about twice as many patients with RLs in zones 1 and 7 with, in addition, a progression tendency to other zones at 24 months. This finding hints at the numerous cases of aseptic loosening seen in the NT group, which can only be attributed to the different surface treatment of these stems.

Stress shielding is an important factor of bone loss in the femoral meta- and diaphysis after uncemented THA [23]. Typically, it takes place during the first 6–12 months, mainly in the Gruen zones 1 and 7 [24–26]. Indeed, the BMD values also stabilized after 6 months in the STD group of the present study. This was, however, not the case for the proximal medial meta- and diaphysis (zones 6 and 7) of the femur in the NT group. The surface treatment is not believed to alter the stiffness of the stem, thus excluding stress-shielding differences as the sole reason of failure. A reduced surface roughness of the NT stem could be attributed to the new surface treatment, where lower profile roughness might impair osseointegration and contribute to the worse outcomes in the present study.

Veldstra et al. [27] conducted a double-blind, randomized controlled study with acetabular cups applying the same surface treatment (acid etching, dry ice blasting). No significant differences in BMD and clinical or radiographic outcomes between the standard and alumina-reduced group were noted at 1 year. Thus, the study was unfortunately prematurely interrupted. However, a longer follow-up would have been of great interest, as in the present study, the median time to revision of NT stems was 18 months (range, 14–50 months).

Indeed, the process of grit blasting followed by acid etching, with HF or other types of acids, is still commonly used as a surface treatment for dental implants to promote osseointegration [28, 29]. A study by Li et al. [30], analyzed the effect of grit-blasted titanium tooth implants treated with HF during acid etching in osteoporotic rats and found a higher surface roughness and better osseointegration than in only grit-blasted implants.

On the other hand, histological results of titanium dental implants in rabbits did not provide evidence that residual alumina particles on the implant surface would affect the osseointegration [14]. Comparably, no significant differences in osseointegration or removal torque force to release the implant were found between grit-blasted implants treated with or without acid-etching 10–12 weeks after implantation in rabbits [31]. Similar results were found in more recent studies, although other surface treatments seem even more promising [32–36].

In the same way that alumina particle remnants from the corundum grit-blasting process could interfere with the osseointegration process of the stem, we wonder whether potential residual traces of HF used for the short-acid etching process could contribute to the poor outcome of these NT stems. In contrast, studies demonstrated that fluoride ions on titanium surfaces resulting from HF treatment have the capacity to increase osteoblast differentiation and gene expression [30].

It is nonetheless important to note that in many in vivo rabbit or rat studies involving dental implants, the follow-up time was between 4 and 12 weeks and the implant had a screw shape [14, 30, 31]. These studies cannot reliably represent the in situ and loading conditions, such as those supported by a hip stem over approximately 20 years. Moreover, the optimal surface roughness in dental implants is lower (1–3 μm) than for hip stems [28, 37–39]. Beside HF, other types of acid were used for surface treatment and the implants were either pure titanium or a titanium alloy [14, 29].

The primary limitation of the present study is the short follow-up time of 24 months for the BMD measurements, as well as the radiological and outcome data. Although the observation period to calculate survival rates was extended up to 50 months, it remains unknown whether further stems failed thereafter. Surgeries were performed more than 10 years ago, and many patients were lost to follow-up, and it is not possible to know how many stems failed thereafter. Secondary, even at the last follow-up of 24 months, about 35% of patients with the NT stem were not included any more. Thirdly, the implant position or the femoral anteversion, which could also influence the outcome after THA, were not assessed postoperatively.

## Conclusions

In this study, rectangular femoral alumina grit-blasted stems with an alumina-reduced surface for THA led to worse clinical outcome scores (WOMAC, HSS) as well as radiographic and DEXA results in terms of BMD, RLs, cortical hypertrophy, and stem subsidence, as compared with standard grit-blasted stems without acid etching and dry ice blasting. Unexpected catastrophic failure rates of at least 41% due to early aseptic loosening were found within a follow-up of 50 months. In the light of these outcome, the NT stem production was discontinued. Considering these results, implants with similar surface treatments are not recommended for use in patients undergoing THA until more conclusive findings concerning this surface treatment are obtained.

## Data Availability

The data that support the findings of this study are available, but restrictions apply to the availability of these data, which were used under license for the current study, and so are not publicly available. Data are however available from the authors upon reasonable request and with permission of Smith + Nephew, Aarau, Switzerland.
